# Triglyceride glucose-body mass index in identifying high-risk groups of pre-diabetes

**DOI:** 10.1186/s12944-021-01594-7

**Published:** 2021-11-14

**Authors:** Chunyuan Jiang, Ruijuan Yang, Maobin Kuang, Meng Yu, Mingchun Zhong, Yang Zou

**Affiliations:** 1 Department of Cardiology, Jiangxi Provincial People’s Hospital Affiliated to Nanchang University, Nanchang, 330006 China; 2 Department of Endocrinology, Jiangxi Provincial People’s Hospital Affiliated to Nanchang University, Nanchang, 330006 China; 3 From the Jiangxi Cardiovascular Research Institute, Jiangxi Provincial People’s Hospital Affiliated to Nanchang University, Nanchang, 330006 China

**Keywords:** Pre-diabetes, TyG-BMI, World Health Organization, Triglyceride glucose-body mass index, American Diabetes Association

## Abstract

**Background:**

Triglyceride glucose-body mass index (TyG-BMI) has been recommended as an alternative indicator of insulin resistance. However, the association between TyG-BMI and pre-diabetes remains to be elucidated.

**Methods:**

More than 100,000 subjects with normal glucose at baseline received follow-up. The main outcome event of concern was pre-diabetes defined according to the diagnostic criteria recommended by the American Diabetes Association (ADA) in 2018 and the World Health Organization (WHO) in 1999. A Cox proportional hazard regression model was used to evaluate the role of TyG-BMI in identifying people at high risk of pre-diabetes.

**Results:**

At a mean observation period of 3.1 years, the incidence of pre-diabetes in the cohort was 3.70 and 12.31% according to the WHO and ADA diagnostic criteria for pre-diabetes, respectively. The multivariate Cox regression analysis demonstrated that TyG-BMI was independently positively correlated with pre-diabetes, and there was a special population dependence phenomenon. Among them, non-obese people, women and people under 50 years old had a significantly higher risk of TyG-BMI-related pre-diabetes (*P*-interaction< 0.05).

**Conclusions:**

These findings suggest that a higher TyG-BMI significantly increases an individual’s risk of pre-diabetes, and this risk is significantly higher in women, non-obese individuals, and individuals younger than 50 years of age.

**Supplementary Information:**

The online version contains supplementary material available at 10.1186/s12944-021-01594-7.

## Background

Pre-diabetes is a subclinical high-risk state that progresses to diabetes and typical diabetes complications. It comprises two characteristics of impaired fasting glucose (IFG) and/or impaired glucose tolerance, and the glucose level is between normal and diabetic [1, 2]. However, at present, there is no unified standard for an IFG-based diagnosis of pre-diabetes [1]. In the standards recommended by the World Health Organization (WHO), the definition of IFG uses fasting plasma glucose (FPG) in the range of 6.1–6.9 mmol/L [3], while the American Diabetes Association (ADA) uses a lower threshold for the definition of IFG (FPG: 5.6–6.9 mmol/L) [4]. In the Chinese population, the prevalence of pre-diabetes diagnosed according to the WHO standard is 15.5%, and that according to the ADA standard is 35.2% [5, 6]. In addition to the high prevalence of pre-diabetes, observational evidence shows that approximately 5–10% of patients with pre-diabetes develop diabetes each year [1, 2]. On the other hand, pre-diabetes significantly increases the risk of macrovascular disease, retinopathy, autonomic neuropathy and kidney disease [7–10]. Therefore, early identification of modifiable risk factors is critical to reducing the long-term medical burden of pre-diabetes.

Previous studies have shown that insulin resistance (IR) is an important feature of the general population developing from normal glucose to pre-diabetes [11, 12], and the current gold standard for IR measurement is the hyperinsulinemic-euglycemic clamp (HEC) technique [13]. However, there are economic, time cost and ethical limitations in using HEC technology to evaluate IR during physical examinations in a large-scale population [14]. Therefore, as an alternative, some effective and inexpensive markers have been developed. Recently, a marker called the triglyceride glucose-body mass index (TyG-BMI) has received much attention. According to the description of Er et al., the ability of TyG-BMI to identify IR is better than that of other IR substitute markers [15]. In addition, some follow-up observational studies found that TyG-BMI has not only excellent discriminating ability in distinguishing IR but also good predictive performance in evaluating hypertension complicated with hyperuricemia, metabolic syndrome (MS), nonalcoholic fatty liver disease (NAFLD) and diabetes [16–19]. However, the longitudinal association between pre-diabetes and TyG-BMI remains to be studied. To address these issues, we retrospectively analysed the relationship between pre-diabetes and TyG-BMI in a large cohort in China according to the diagnostic criteria of pre-diabetes recommended by the WHO and ADA.

## Methods

### Study design and subjects

**Fig. 1:**
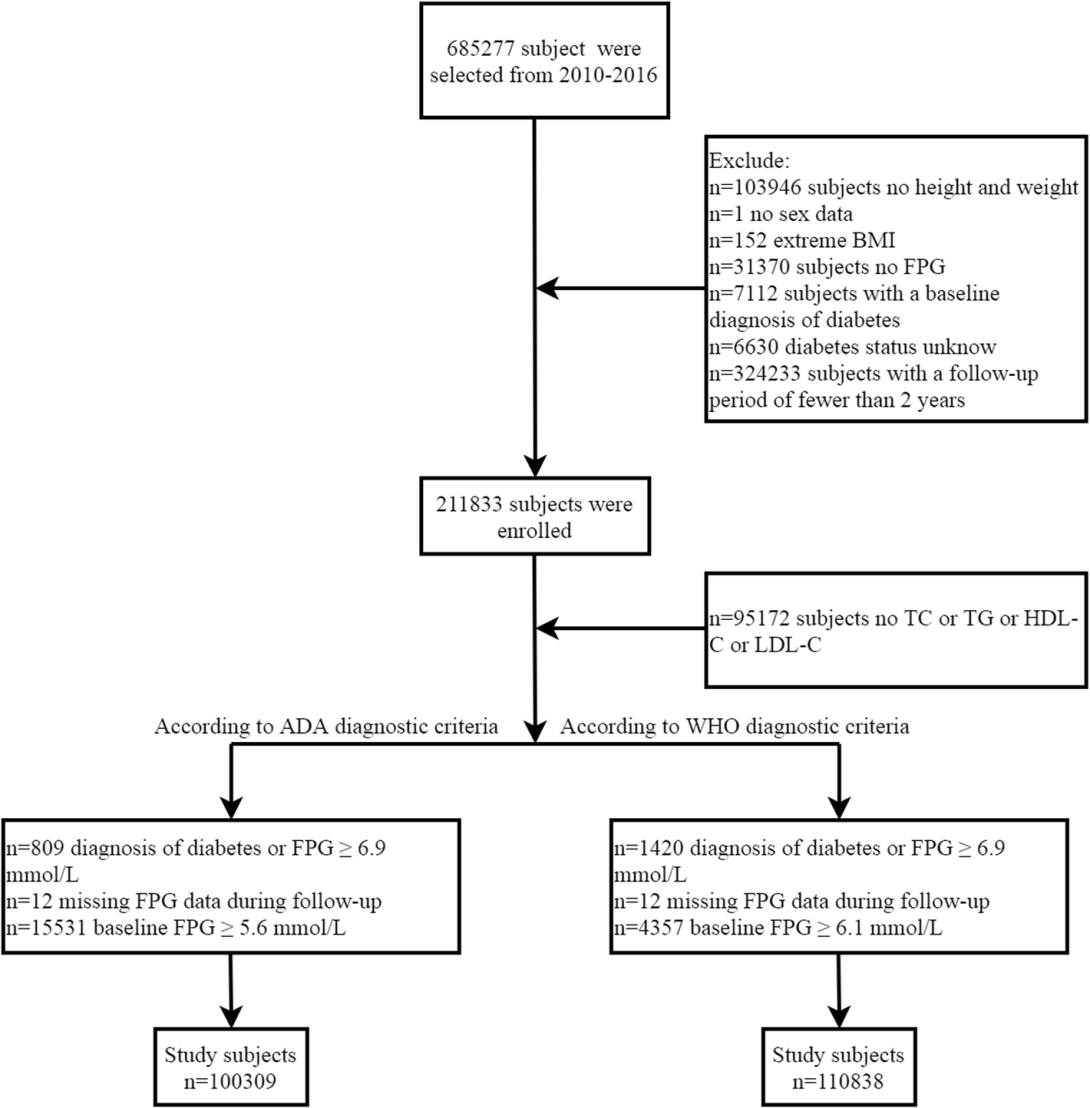
Flow-chart of study selection process

This is a post-hoc analysis of a large longitudinal cohort in China established by Rich Healthcare Group. The dataset of the analysis has been stored in the Dryad database by Chen et al. With reference to the terms of service of the database, researchers can reasonably apply data and indicate the source of data packets based on different research hypotheses [20]. In a previous study, Chen et al. analysed the association between age, body mass index (BMI) and diabetes [21], and the detailed study design was also described in a previous study [21]. In short, the original cohort of the study included all adults who underwent health examinations at Rich Healthcare Group in 11 major cities in China from 2010 to 2016. Subjects with at least two consecutive follow-up records were included. Subjects with incomplete measurements of height, weight and sex, unmeasured FPG, and extreme BMI values (subjects with BMI > 55 kg/m^2^ or < 15 kg/m^2^ were excluded, and the BMI range of 15–52.7 kg/^2^ was allowed for inclusion in the study); diagnosed with diabetes at the baseline visit; followed up for less than 2 years and whose diabetes status could not be determined during follow-up were excluded. Finally, Chen et al. analysed the data of 211,833 people who underwent a physical examination. Our current study was a post-hoc analysis of the dataset shared by Chen et al., and we added the following exclusion criteria according to the diagnostic criteria of ADA and WHO for pre-diabetes: (1) subjects with incomplete lipid parameters; (2) subjects with FPG ≥ 6.1 mmol/L (according to WHO diagnostic criteria) or ≥ 5.6 mmol/L (according to ADA diagnostic criteria) at the baseline visit; (3) subjects self-reporting diagnosed diabetes or FPG > 6.9 during follow-up; and (4) subjects with missing FPG information during follow-up. Finally, 110,838 subjects could be used for analysis according to the diagnostic criteria of WHO for pre-diabetes and 100,309 subjects could be used for analysis according to the diagnostic criteria of ADA for pre-diabetes. Figure 1 shows a flow chart for study population inclusion. As the ethics committee of Rich Healthcare Group had authorized the previous study, the ethics committee of Jiangxi Provincial People’s Hospital exempted the repeated application for ethical approval for this study (ethical review No. 2021–067).

### Data collection

Trained health care workers recorded the baseline information of the subjects through standard questionnaires, including height, weight, age, sex, blood pressure, smoking and drinking history, family history and other biochemical indicators measured by physical examination. Height and weight measurements required subjects to take off their shoes and wear only lightweight clothes, with measurements accurate to 0.1 kg and 0.1 cm. Analytical samples of FPG and other biochemical parameters were obtained at least 10 h after fasting and determined by a Beckman 5800 biochemical analyser. The same procedure was performed during the first visit and follow-up.

### TyG-BMI calculation

TyG-BMI = BMI × TyG index, where BMI = weight/height^2^, and the TyG index = Ln [1/2FPG (mg/dL) × TG (mg/dL)] [15].

### Diagnosis of pre-diabetes

In this study, we referred to the diagnostic criteria of IFG-based pre-diabetes recommended by the WHO in 1999 and ADA in 2018 [3, 4]; the WHO’s IFG-based pre-diabetes diagnostic criteria were defined as FPG levels from 6.1 to 6.9 mmol/L [3], and the ADA’s IFG-based pre-diabetes diagnostic criteria were defined as FPG levels from 5.6 to 6.9 mmol/L [4].

### Statistical analysis

In this study, two sets of data were obtained after inclusion and exclusion according to the diagnostic criteria of pre-diabetes recommended by the WHO and ADA. The same steps were performed in both sets of data for all analyses. The statistical software for analysing the data were R language 3.4.3 and Empower (R) 2.20. The following outlines the specific statistical analysis in this study.

1. Description of baseline characteristics: First, the quartile of TyG-BMI was calculated by a quantile function; then, the baseline data of subjects were stratified based on TyG-BMI quartiles and whether pre-diabetes was diagnosed; finally, the differences between groups were evaluated using a t test, Kruskal–Wallis test, chi-squared test or one-way analysis of variance. Variables were described as percentages (%), medians (quartile intervals) or means (standard deviations: SD).
2. Association analysis: A Cox proportional hazard regression model was used to evaluate the relationship between pre-diabetes and TyG-BMI, with hazard ratios (HR) and 95% confidence intervals (CI) calculated. Before the model was established, the collinearity used by the variance inflation factor to evaluate covariates was calculated (Supplementary Table 1) [22]. Then, the cumulative incidence of pre-diabetic events with time was calculated by the Kaplan–Meier (KM) method, and whether it conformed to the proportional risk hypothesis of establishing the Cox model was determined by observing the KM curve corresponding to the TyG-BMI quartile. After following the above premise, we referred to the Strengthening the Reporting of Observational Studies in Epidemiology statement to implement the model adjustment strategy [23]. Model I adjusted for variables that had an impact of > 10% on the risk of pre-diabetes associated with TyG-BMI [24]. Model II adjusted for covariates related to pre-diabetes + Model I. Model III adjusted for all non-collinear variables. The trend *P* for TyG-BMI and pre-diabetes risk was examined in all three models by incorporating the TyG-BMI quartiles into the model as a continuous variable for analysis. Additionally, we also performed a receiver operating characteristic (ROC) curve analysis and calculated the best threshold and the area under the ROC curve (AUROC) for TyG-BMI for predicting pre-diabetes.
3. Subgroup analysis: This study conducted a stratified analysis based on the family history of diabetes, BMI, sex and age. Among them, the stratification of age refers to the research of Chen et al., and the classification was made according to decades [21]; the stratification of BMI refers to the standard recommended by the Chinese Obesity working Group, with 24 kg/m^2^ as the cut-off point for overweight [25]. The stratified analysis was conducted through a Cox regression model, the differences between layers were compared by the likelihood ratio test, and the interaction was evaluated.

## Results

### Clinical characteristics of subjects in the TyG-BMI quartile groups

The dataset for inclusion and exclusion according to the WHO diagnostic criteria for pre-diabetes included 110,838 people (average age of 43.5 years old, 52.98% men, and 47.02% women). The dataset for inclusion and exclusion according to the ADA diagnostic criteria for pre-diabetes included 100,309 people (average age of 42.9 years, 51.97% men, and 48.03% women). In the two datasets included on the subjects according to ADA and WHO standards, we found significant differences in the clinical characteristics of the subjects among the quartiles of TyG-BMI (Table 1). Among them, the general measurement parameters, such as blood pressure, height, BMI and weight, in the group with high TyG-BMI levels were higher than those in the group with low TyG-BMI levels. In addition, subjects with high levels of TyG-BMI generally had a higher proportion of men and older ages, with higher levels of FPG, creatinine (Cr), total cholesterol (TC), aspartate aminotransferase (AST), low density lipoprotein cholesterol (LDL-C), blood urea nitrogen (BUN), triglyceride (TG) and alanine aminotransferase (ALT) and lower levels of high density lipoprotein cholesterol (HDL-C).

**Table 1:** Baseline characteristics based on TyG-BMI grouping of subjects enrolled according to ADA and WHO diagnostic criteria for pre-diabetes

	TyG-BMI quartile				
According to WHO diagnostic criteria					
	Q1(96.68–168.15)	Q2(168.15–192.09)	Q3(192.09–218.70)	Q4(218.70–477.08)	*P*-value
No. of subjects	27,710	27,709	27,709	27,710	
Age, years	35.00 (31.00–43.00)	40.00 (33.00–51.00)	44.00 (35.00–55.00)	45.00 (36.00–56.00)	< 0.001
Sex					< 0.001
Men	7394 (26.68%)	12,526 (45.21%)	17,684 (63.82%)	21,118 (76.21%)	
Women	20,316 (73.32%)	15,183 (54.79%)	10,025 (36.18%)	6592 (23.79%)	
Height, cm	164.16 (7.50)	165.43 (8.30)	167.12 (8.48)	168.41 (8.31)	< 0.001
Weight, kg	52.76 (6.02)	60.48 (6.93)	67.47 (7.70)	77.44 (10.06)	< 0.001
BMI, kg/m^2^	19.54 (1.37)	22.04 (1.19)	24.10 (1.30)	27.24 (2.32)	< 0.001
SBP, mmHg	110.65 (13.57)	116.14 (15.08)	121.46 (15.63)	126.96 (16.30)	< 0.001
DBP, mmHg	69.22 (9.19)	72.06 (9.83)	75.64 (10.35)	79.59 (11.06)	< 0.001
FPG, mmol/L	4.69 (0.51)	4.84 (0.52)	4.94 (0.52)	5.06 (0.53)	< 0.001
TC, mmol/L	4.45 (0.79)	4.67 (0.84)	4.88 (0.88)	5.09 (0.91)	< 0.001
TG, mmol/L	0.68 (0.52–0.88)	0.93 (0.72–1.20)	1.27 (0.97–1.68)	1.90 (1.40–2.64)	< 0.001
HDL-C, mmol/L	1.50 (1.30–1.70)	1.39 (1.22–1.60)	1.31 (1.13–1.50)	1.22 (1.05–1.41)	< 0.001
LDL-C, mmol/L	2.45 (2.12–2.85)	2.64 (2.27–3.08)	2.81 (2.40–3.27)	2.89 (2.46–3.36)	< 0.001
ALT, U/L	13.00 (10.30–17.00)	15.90 (12.00–22.00)	20.00 (15.00–28.00)	27.50 (19.10–40.40)	< 0.001
AST, U/L	20.00 (17.00–23.00)	21.00 (18.00–25.00)	22.50 (19.00–27.00)	25.00 (21.00–30.80)	< 0.001
BUN, mmol/L	4.30 (3.60–5.10)	4.49 (3.77–5.30)	4.66 (3.95–5.48)	4.75 (4.04–5.55)	< 0.001
Cr, umol/L	60.70 (53.60–71.50)	66.40 (56.40–79.20)	73.00 (61.00–83.40)	76.20 (65.90–85.80)	< 0.001
TyG index	7.84 (0.38)	8.19 (0.39)	8.52 (0.42)	8.97 (0.50)	< 0.001
Family history of diabetes	571 (2.06%)	687 (2.48%)	583 (2.10%)	617 (2.23%)	0.004
Smoking status					< 0.001
Non	691 (2.49%)	1131 (4.08%)	1719 (6.20%)	2575 (9.29%)	
Past	127 (0.46%)	234 (0.84%)	401 (1.45%)	474 (1.71%)	
Current	6078 (21.93%)	5962 (21.52%)	5899 (21.29%)	5628 (20.31%)	
Not recorded	20,814 (75.11%)	20,382 (73.56%)	19,690 (71.06%)	19,033 (68.69%)	
Drinking status					< 0.001
Non	63 (0.23%)	120 (0.43%)	225 (0.81%)	376 (1.36%)	
Past	623 (2.25%)	1058 (3.82%)	1540 (5.56%)	1993 (7.19%)	
Current	6210 (22.41%)	6149 (22.19%)	6254 (22.57%)	6308 (22.76%)	
Not recorded	20,814 (75.11%)	20,382 (73.56%)	19,690 (71.06%)	19,033 (68.69%)	
According to ADA diagnostic criteria					
	Q1(96.68–166.56)	Q2(166.56–189.88)	Q3(189.88–216.05)	Q4(216.06–477.08)	
No. of subjects	25,077	25,077	25,077	25,078	
Age, years	35.00 (31.00–42.00)	39.00 (33.00–49.00)	43.00 (35.00–54.00)	44.00 (36.00–55.00)	< 0.001
Sex					< 0.001
Men	6500 (25.92%)	10,892 (43.43%)	15,657 (62.44%)	19,081 (76.09%)	
Women	18,577 (74.08%)	14,185 (56.57%)	9420 (37.56%)	5997 (23.91%)	
Height, cm	164.13 (7.46)	165.29 (8.24)	167.06 (8.49)	168.46 (8.30)	< 0.001
Weight, kg	52.50 (5.93)	59.99 (6.83)	67.00 (7.64)	77.05 (10.01)	< 0.001
BMI, kg/m^2^	19.45 (1.35)	21.90 (1.17)	23.94 (1.29)	27.08 (2.30)	< 0.001
SBP, mmHg	110.30 (13.41)	115.25 (14.83)	120.60 (15.33)	126.16 (16.12)	< 0.001
DBP, mmHg	69.05 (9.15)	71.61 (9.76)	75.19 (10.22)	79.16 (11.06)	< 0.001
FPG, mmol/L	4.65 (0.48)	4.76 (0.47)	4.83 (0.46)	4.91 (0.44)	< 0.001
TC, mmol/L	4.44 (0.79)	4.65 (0.83)	4.85 (0.87)	5.06 (0.91)	< 0.001
TG, mmol/L	0.67 (0.52–0.86)	0.90 (0.70–1.18)	1.23 (0.95–1.62)	1.87 (1.37–2.57)	< 0.001
HDL-C, mmol/L	1.50 (1.30–1.70)	1.40 (1.23–1.60)	1.31 (1.14–1.50)	1.22 (1.05–1.41)	< 0.001
LDL-C, mmol/L	2.44 (2.11–2.84)	2.63 (2.26–3.05)	2.79 (2.37–3.24)	2.87 (2.45–3.34)	< 0.001
ALT, U/L	13.00 (10.30–17.00)	15.40 (11.90–21.30)	19.60 (14.50–27.80)	27.00 (19.00–40.00)	< 0.001
AST, U/L	20.00 (17.00–23.00)	21.00 (18.00–24.60)	22.00 (19.00–26.60)	24.90 (20.90–30.20)	< 0.001
BUN, mmol/L	4.28 (3.60–5.08)	4.45 (3.74–5.23)	4.61 (3.91–5.42)	4.71 (4.01–5.50)	< 0.001
Cr, ummol/L	60.50 (53.40–71.00)	65.90 (56.00–78.80)	72.70 (60.80–83.00)	76.20 (65.90–85.60)	< 0.001
TyG index	7.82 (0.38)	8.15 (0.38)	8.47 (0.41)	8.91 (0.50)	< 0.001
Family history of diabetes	513 (2.05%)	607 (2.42%)	530 (2.11%)	558 (2.23%)	0.025
Smoking status					< 0.001
Non	614 (2.45%)	985 (3.93%)	1486 (5.93%)	2264 (9.03%)	
Past	110 (0.44%)	198 (0.79%)	349 (1.39%)	433 (1.73%)	
Current	5441 (21.70%)	5372 (21.42%)	5301 (21.14%)	5085 (20.28%)	
Not recorded	18,912 (75.42%)	18,522 (73.86%)	17,941 (71.54%)	17,296 (68.97%)	
Drinking status					< 0.001
Non	56 (0.22%)	100 (0.40%)	177 (0.71%)	306 (1.22%)	
Past	530 (2.11%)	928 (3.70%)	1323 (5.28%)	1767 (7.05%)	
Current	5579 (22.25%)	5527 (22.04%)	5636 (22.47%)	5709 (22.76%)	
Not recorded	18,912 (75.42%)	18,522 (73.86%)	17,941 (71.54%)	17,296 (68.97%)	

Continuous variables were summarized as mean (SD) or medians (quartile interval), the differences among quartiles were evaluated by one-way analysis of variance and Tukey’s HSD test or the Kruskal–Wallis test and Steel–Dwass test. After making a pairwise comparison between the quintiles, the results showed that there were significant differences among all groups (*P* < 0.05). Categorical variables were displayed as percentage (%)

*Abbreviations*: *ADA* American Diabetes Association, *WHO* World Health Organization, *BMI* Body mass index, *SBP* systolic blood pressure, *DBP* diastolic blood pressure, *FPG* fasting plasma glucose, *TG* triglyceride, *TyG* the triglyceride-glucose index, *TyG-BMI* triglyceride glucose-body mass index, *TC* total cholesterol, *LDL-C* low-density lipid cholesterol, *ALT* alanine aminotransferase, *AST* aspartate aminotransferase, *BUN* blood urea nitrogen, *Cr* creatinine

### Clinical characteristics of pre-diabetes

**Fig. 2:**
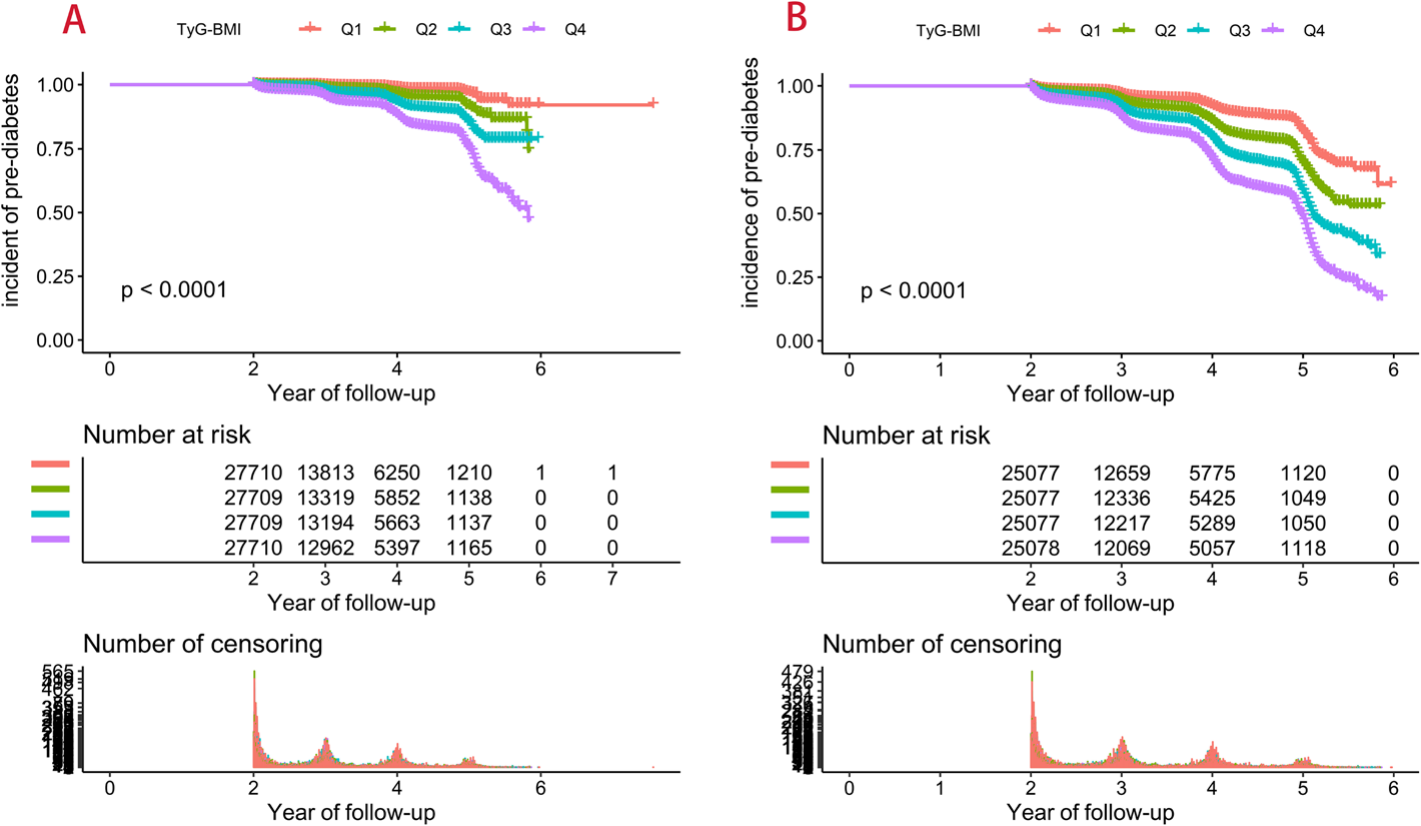
Kaplan–Meier curve for pre-diabetes (**A**: according to WHO diagnostic criteria; **B**: according to ADA diagnostic criteria). TyG-BMI: triglyceride glucose-body mass index; WHO: World Health Organization; ADA: American Diabetes Association

At a mean observation period of 3.1 years, the incidence of pre-diabetes in the cohort was 3.70 and 12.31% according to WHO and ADA diagnostic criteria for pre-diabetes. The KM analysis showed a gradual increase in the cumulative incidence of pre-diabetes between the TyG-BMI quartile group (Fig. 2). Table 2 shows the clinical characteristics of individuals in the pre-diabetes group and the non-pre-diabetes group, and the results of the analysis of the two datasets were similar. Individuals with pre-diabetes were generally older; were more often male; had higher weight, BMI, SBP, and DBP; had higher levels of Cr, BUN, AST, ALT, LDL-C, TG, TC, and TyG-BMI; had lower levels of HDL-C; and were less likely to maintain smoking and drinking habits. These results suggest that patients with pre-diabetes appear to have a trend of metabolic disorders in an earlier period.

**Table 2:** Baseline characteristics of the pre-diabetes and non-pre-diabetes groups

	WHO		*P*-value	ADA		*P*-value
	Non-Pre-diabetes	Pre-diabetes		Non-Pre-diabetes	Pre-diabetes	
No. of subjects	106,735	4103		87,957	12,352	
Age, years	40.00 (33.00–51.00)	53.00 (44.00–62.00)	< 0.001	39.00 (33.00–49.00)	48.00 (37.00–59.00)	< 0.001
Sex			< 0.001			< 0.001
Men	56,105 (52.56%)	2617 (63.78%)		44,395 (50.47%)	7735 (62.62%)	
Women	50,630 (47.44%)	1486 (36.22%)		43,562 (49.53%)	4617 (37.38%)	
Height, cm	166.28 (8.30)	166.38 (8.57)	0.430	166.17 (8.28)	166.69 (8.43)	< 0.001
Weight, kg	64.32 (11.93)	70.09 (12.30)	< 0.001	63.59 (11.81)	68.02 (11.91)	< 0.001
BMI, kg/m^2^	23.15 (3.22)	25.21 (3.28)	< 0.001	22.91 (3.17)	24.38 (3.25)	< 0.001
SBP, mmHg	118.38 (16.11)	129.91 (18.46)	< 0.001	117.08 (15.61)	125.17 (17.52)	< 0.001
DBP, mmHg	73.91 (10.76)	79.85 (11.62)	< 0.001	73.21 (10.57)	77.61 (11.38)	< 0.001
FPG, mmol/L	4.86 (0.53)	5.38 (0.48)	< 0.001	4.75 (0.47)	5.03 (0.40)	< 0.001
TC, mmol/L	4.76 (0.89)	5.05 (0.93)	< 0.001	4.72 (0.88)	4.92 (0.90)	< 0.001
TG, mmol/L	1.08 (0.74–1.60)	1.50 (1.00–2.20)	< 0.001	1.02 (0.72–1.52)	1.30 (0.90–1.94)	< 0.001
HDL-C, mmol/L	1.35 (1.17–1.57)	1.32 (1.12–1.52)	< 0.001	1.36 (1.17–1.58)	1.32 (1.14–1.53)	< 0.001
LDL-C, mmol/L	2.68 (2.28–3.14)	2.83 (2.42–3.33)	< 0.001	2.66 (2.26–3.11)	2.77 (2.36–3.25)	< 0.001
ALT,U/L	17.90 (12.90–26.90)	22.00 (16.00–33.00)	< 0.001	17.10 (12.40–26.00)	21.00 (14.70–31.00)	< 0.001
AST,U/L	22.00 (18.40–26.20)	23.80 (20.00–28.00)	< 0.001	21.60 (18.10–26.00)	23.00 (19.00–28.00)	< 0.001
BUN, mmol/L	4.53 (3.81–5.35)	4.88 (4.13–5.72)	< 0.001	4.50 (3.79–5.30)	4.73 (4.00–5.55)	< 0.001
Cr, umol/L	69.10 (57.70–81.00)	73.00 (62.00–84.00)	< 0.001	68.30 (57.00–80.30)	73.00 (61.30–83.90)	< 0.001
TyG index	8.36 (0.59)	8.77 (0.61)	< 0.001	8.30 (0.57)	8.58 (0.59)	< 0.001
TyG-BMI	194.49 (35.64)	221.76 (36.36)	< 0.001	191.08 (34.69)	209.92 (35.77)	< 0.001
Family history of diabetes	2358 (2.21%)	100 (2.44%)	0.330	1898 (2.16%)	310 (2.51%)	0.013
Smoking status			< 0.001			< 0.001
Non	5825 (5.46%)	291 (7.09%)		4509 (5.13%)	840 (6.80%)	
Past	1189 (1.11%)	47 (1.15%)		935 (1.06%)	155 (1.25%)	
Current	22,902 (21.46%)	665 (16.21%)		18,877 (21.46%)	2322 (18.80%)	
Not recorded	76,819 (71.97%)	3100 (75.55%)		63,636 (72.35%)	9035 (73.15%)	
Drinking status			< 0.001			< 0.001
Non	736 (0.69%)	48 (1.17%)		539 (0.61%)	100 (0.81%)	
Past	5025 (4.71%)	189 (4.61%)		3944 (4.48%)	604 (4.89%)	
Current	24,155 (22.63%)	766 (18.67%)		19,838 (22.55%)	2613 (21.15%)	
Not recorded	76,819 (71.97%)	3100 (75.55%)		63,636 (72.35%)	9035 (73.15%)	

Abbreviations as in Table 1

### Association of TyG-BMI with pre-diabetes

**Fig. 3:**
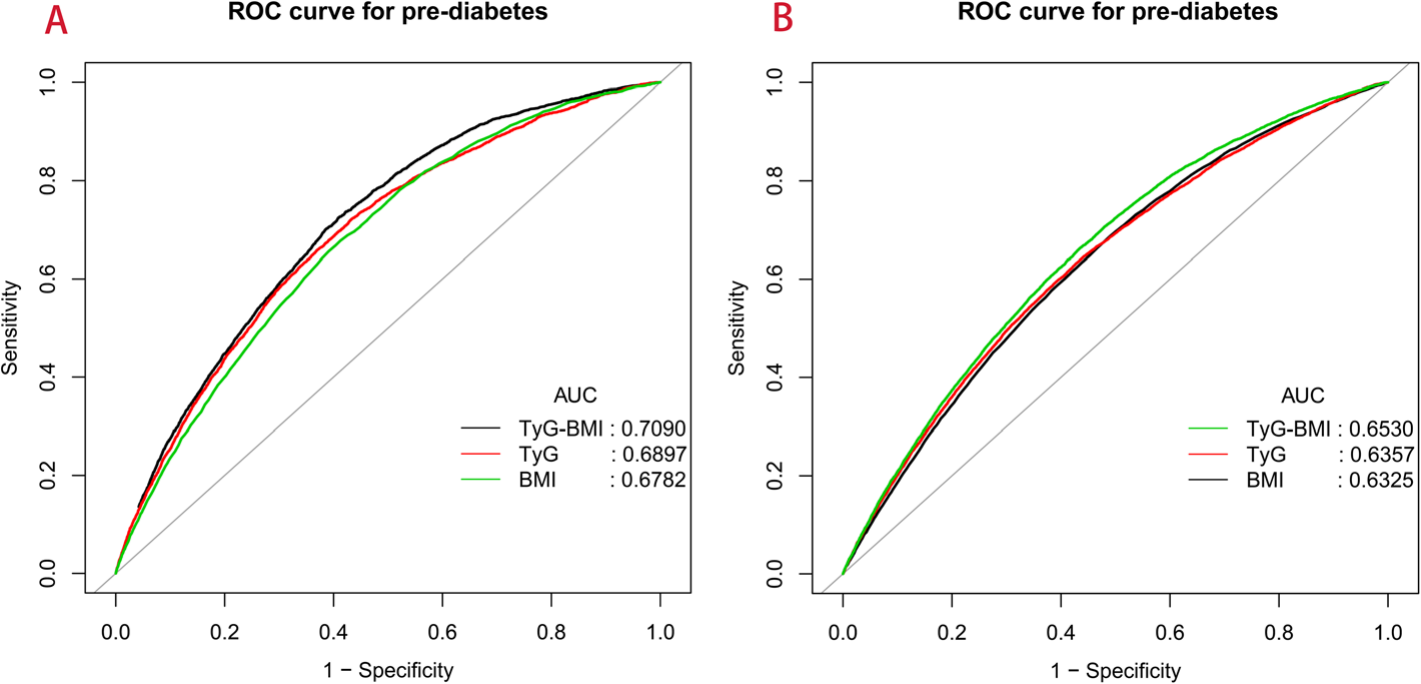
Receiver operating characteristic curve analyses for predicting pre-diabetes (**A**: according to WHO diagnostic criteria; **B**: according to ADA diagnostic criteria). AUC: area under the curve; BMI: body mass index, TyG index: triglyceride-glucose index, TyG-BMI: triglyceride glucose-body mass index

The association of TyG-BMI with pre-diabetes was evaluated by a Cox regression model (Model I-III, Table 3). Regardless of the WHO or ADA diagnostic criteria for pre-diabetes, TyG-BMI was positively correlated with pre-diabetes in all three models (*P*-trend< 0.0001). Even after adjusting for all non-collinear variables, the regression coefficient of pre-diabetes risk corresponding to TyG-BMI was only slightly decreased (ADA: HR: 1.23, 95% CI: 1.18–1.27; WHO: HR 1.40, 95% CI: 1.32–1.47). Additionally, an ROC analysis was performed to evaluate the predictive value of TyG-BMI for pre-diabetes (Fig. 3). Table 4 shows the AUROC and best thresholds used by TyG-BMI, the TyG index and BMI to predict pre-diabetes. In the two datasets, the AUROC of TyG-BMI was the largest, showing moderate predictive performance for pre-diabetes.

**Table 3:** Cox regression analyses for the association between TyG-BMI and incident pre-diabetes in different models

TyG-BMI	HR, 95% CI			
	Unadjusted Model	Model I	Model II	Model III
WHO				
Multivariable Analysis (HR Per SD increase)	1.89 (1.85, 1.94)	1.42 (1.38, 1.47)	1.39 (1.32, 1.47)	1.40 (1.32, 1.47)
TyG-BMI quartile				
Q1	Ref	Ref	Ref	Ref
Q2	2.56 (2.21, 2.97)	1.60 (1.38, 1.86)	1.59 (1.30, 1.96)	1.60 (1.30, 1.96)
Q3	4.83 (4.21, 5.54)	2.18 (1.89, 2.52)	2.17 (1.78, 2.65)	2.17 (1.78, 2.65)
Q4	8.93 (7.84, 10.19)	2.96 (2.57, 3.42)	2.77 (2.25, 3.40)	2.77 (2.26, 3.41)
*P*-trend	< 0.0001	< 0.0001	< 0.0001	< 0.0001
ADA				
Multivariable Analysis (HR Per SD increase)	1.54 (1.52, 1.56)	1.27 (1.24, 1.29)	1.23 (1.18, 1.27)	1.23 (1.18, 1.27)
TyG-BMI quartile				
Q1	Ref	Ref	Ref	Ref
Q2	1.74 (1.63, 1.86)	1.34 (1.25, 1.43)	1.36 (1.23, 1.50)	1.36 (1.23, 1.49)
Q3	2.66 (2.50, 2.83)	1.70 (1.59, 1.81)	1.56 (1.41, 1.71)	1.56 (1.41, 1.71)
Q4	3.86 (3.64, 4.10)	2.03 (1.90, 2.17)	1.82 (1.63, 2.02)	1.81 (1.63, 2.02)
*P*-trend	< 0.0001	< 0.0001	< 0.0001	< 0.0001

*Abbreviations*: *TyG-BMI* triglyceride glucose-body mass index, *HR* hazard ratios, *CI* confidence, *ADA* American Diabetes Association, *WHO* World Health Organization

WHO: Model I adjusted for age, SBP, DBP, FPG, ALT and HDL-C; Model II adjusted for age, sex, SBP, DBP, FPG, TG, HDL-C, LDL-C, ALT, AST, BUN, Cr, drinking status and smoking status. Model III adjusted for age, sex, SBP, DBP, FPG, TG, HDL-C, LDL-C, ALT, AST, BUN, Cr, drinking status, smoking status, height and family history of diabetes

ADA: Model I adjusted for age, SBP, DBP, FPG, ALT and HDL-C; Model II adjusted for age, sex, SBP, DBP, FPG, TG, HDL-C, LDL-C, ALT, AST, BUN, Cr, smoking status, height and drinking status. Model II adjusted for age, sex, SBP, DBP, FPG, TG, HDL-C, LDL-C, ALT, AST, BUN, Cr, smoking status, height, drinking status and family history of diabetes

**Table 4:** Areas under the receiver operating characteristic curves for TyG-BMI, TyG index and BMI in identifying pre-diabetes

	AUROC	95% CI low	95% CI upp	Best threshold	Specificity	Sensitivity
WHO						
TyG-BMI	0.7090	0.7015	0.7165	200.0325	0.5913	0.7243
TyG index	0.6897	0.6817	0.6977	8.5305	0.6314	0.6573
BMI	0.6782	0.6703	0.6860	23.6950	0.5865	0.6785
ADA						
TyG-BMI	0.6530	0.6480	0.6580	193.4081	0.5657	0.6625
TyG index	0.6357	0.6306	0.6409	8.3711	0.5727	0.6319
BMI	0.6325	0.6274	0.6375	22.7950	0.5101	0.6870

*Abbreviations*: *AUROC* Areas under the receiver operating characteristic curves, *BMI* body mass index, *TyG index* triglyceride glucose index, *TyG-BMI* triglyceride glucose-body mass index, *ADA* American Diabetes Association, *WHO* World Health Organization

### Subgroup analysis

To explore whether there are differences in the risk for TyG-BMI-related pre-diabetes among different populations, a stratified analysis was also conducted. As shown in Table 5, higher levels of TyG-BMI among women, people under the age of 50, and non-obese people suggest a higher risk of developing pre-diabetes (*P*-interaction< 0.05). In addition, although people with a family history of diabetes in the ADA group appeared to have a higher risk of developing pre-diabetes in the stratified analysis, further interaction tests suggested no significant difference (*P*-interaction> 0.05).

**Table 5:** Stratified associations between TyG-BMI and pre-diabetes by age, sex, BMI and family history of diabetes

Subgroup	WHO	*P*-value	*P*-interaction	ADA	*P*-value	*P*-interaction
	adjusted HR (95% CI)			adjusted HR (95% CI)		
Age (years)			< 0.0001			< 0.0001
18–29	1.50 (1.29, 1.75)	< 0.0001		1.34 (1.25, 1.43)	< 0.0001	
30–39	1.62 (1.52, 1.73)	< 0.0001		1.35 (1.31, 1.40)	< 0.0001	
40–49	1.58 (1.48, 1.69)	< 0.0001		1.35 (1.31, 1.40)	< 0.0001	
50–59	1.37 (1.29, 1.45)	< 0.0001		1.23 (1.18, 1.28)	< 0.0001	
60–69	1.21 (1.12, 1.29)	< 0.0001		1.08 (1.03, 1.13)	0.0031	
≥ 70	1.19 (1.08, 1.32)	< 0.0001		1.10 (1.02, 1.17)	0.0086	
Sex			0.0182			< 0.0001
Women	1.49 (1.41, 1.57)	< 0.0001		1.40 (1.35, 1.44)	< 0.0001	
Men	1.38 (1.33, 1.44)	< 0.0001		1.18 (1.15, 1.20)	< 0.0001	
BMI (kg/m^2^)			< 0.0001			< 0.0001
< 18.5	1.43 (0.47, 4.32)	0.5252		1.93 (1.25, 2.99)	0.0030	
≥ 18.5, < 24	1.84 (1.66, 2.04)	< 0.0001		1.51 (1.44, 1.59)	< 0.0001	
≥ 24, < 28	1.34 (1.22, 1.46)	< 0.0001		1.26 (1.19, 1.33)	< 0.0001	
≥ 28	1.30 (1.18, 1.43)	< 0.0001		1.15 (1.07, 1.23)	0.0001	
Family history of diabetes			0.0625			0.4701
Yes	1.20 (0.99, 1.44)	0.0576		1.31 (1.19, 1.45)	< 0.0001	
No	1.43 (1.38, 1.48)	< 0.0001		1.27 (1.24, 1.29)	< 0.0001	

*Abbreviations*: *BMI* body mass index, *TyG-BMI* triglyceride glucose-body mass index, *HR* hazard ratios, *CI* confidence, *ADA* American Diabetes Association, *WHO* World Health Organization

The model adjusted for age, SBP, DBP, FPG, ALT and HDL-C;

Note: For age stratified analysis, the stratification variable was omitted from the model

## Discussion

This large retrospective cohort study demonstrated that over time higher TyG-BMI levels in healthy adults with normal glucose were positively correlated with new-onset pre-diabetes. After further adjusting for age, sex, lifestyle factors, arterial blood pressure, and serological factors, the positive association between pre-diabetes and TyG-BMI remained stable. Additionally, the ROC analysis showed that TyG-BMI also had a moderate predictive value for pre-diabetes.

In recent years, with the advancement of urbanization, the nutrient intake ratio of residents has undergone a rapid change, accompanied by a significant decline in physical activity, and the glucose level of the global population is gradually rising [26]. From 1980 to 2008, the FPG of the whole population increased by approximately 0.1 mmol/L, including 5.4 mmol/L in women and 5.5 mmol/L in men [27]. The incidence of pre-diabetes is increasing worldwide, and in China the prevalence of pre-diabetes according to the ADA diagnostic criteria has exceeded 35% [1, 6].

At present, the diagnosis of pre-diabetes in China mostly refers to the standard recommended by ADA, and some studies refer to the diagnostic standard of the WHO. The prevalence of pre-diabetes diagnosed according to the ADA criteria is also higher due to its lower diagnosis threshold for IFG-based pre-diabetes [7–10]. Considering that the definition of pre-diabetes is not uniform, a high threshold may lead to missed diagnosis in some high-risk individuals, while a lower threshold can easily lead to overdiagnosis. Therefore, in the same cohort, the diagnostic criteria of the WHO and ADA were referenced in this study to evaluate the association of pre-diabetes with TyG-BMI and to further verify the stability of the association between the two. At an average observation period of 3.1 years, there was a significant difference in the number of pre-diabetes diagnoses according to the two criteria, with an incidence of pre-diabetes diagnosed according to the WHO criteria of 3.70% and an incidence of pre-diabetes diagnosed according to the ADA criteria of 12.31%. The results of these differences were similar to those of previous surveys [5, 6].

### Comparisons with other studies and what does the current work add to the existing knowledge

The triglyceride glucose (TyG) index is a combined marker containing TG and FPG, and it has been widely studied in the early period and is regarded as an alternative marker of IR [28, 29]. In a recent study by Wen et al., the TyG index was found to also identify people at risk of pre-diabetes [30]. Their findings further suggest that IR is closely related to pre-diabetes. BMI is the simplest anthropometric indicator, and it is often used to assess obesity and the risk of metabolic diseases [31]. TyG-BMI is a newly developed obesity-related parameter in recent years. It is the product of BMI and the TyG index. In the earliest related study of TyG-BMI, Er et al. found that TyG-BMI is a better predictor of IR than the TyG index, BMI, traditional lipids, lipid ratios, the visceral obesity index, the visceral adiposity index, lipid accumulation products, and adipose factors [15]. Recently, more evidence has shown that TyG-BMI is closely related to adverse metabolic characteristics, including abnormal serum uric acid, abnormal blood pressure, dyslipidemia and abnormal glucose [16–19]. In March 2021, Raimi et al. analysed the association of TyG-BMI with MS. They noted that the product of BMI and the TyG index improved the ability to identify and predict MS (AUROC = 0.838) [16]. In May, another study found that TyG-BMI also had a moderate ability to distinguish between hypertension and hyperuricemia [17]. Apart from that, several observational studies further found that TyG-BMI has the potential to predict the risk of NAFLD and diabetes, especially in young and middle-aged people and non-obese people [18, 19]. These TyG-BMI-related results suggest that this parameter has the potential to be used as a marker of metabolic diseases. However, the current research on the association of pre-diabetes with TyG-BMI is limited, and there are only two related studies. Zheng et al. first found that TyG-BMI can be used to assess the pre-diabetes risk of first-degree relatives of diabetic patients [32], and a subsequent study in India found a similar association in the general population [33]. Although these two studies have revealed an association between TyG-BMI and pre-diabetes, there are still some limitations in their studies. (1) Follow-up data were not included in their study, so a longitudinal association could not be analysed. (2) Their study did not further analyse the differences in the relationship between the two groups in different populations. (3) Their sample size is relatively small (166 and 1544). (4) The diagnostic criteria for pre-diabetes were different in their study. To further explore the longitudinal association of pre-diabetes with TyG-BMI, this study included the sample data of more than 100,000 subjects and referred to the diagnostic criteria of the ADA and WHO at the same time. The results confirmed their conclusions and further found that the association between the two was significantly different in different populations. Additionally, we note that the risk factors for pre-diabetes, diabetes and cardiovascular disease in the Asian population are similar to those of other ethnic groups [34, 35]. These findings further imply that TyG-BMI may be equally useful in cardiovascular disease risk assessment.

New evidence in this study suggests that in people with a family history of diabetes, the relationship between pre-diabetes and TyG-BMI based on ADA diagnostic criteria is significant. This result is consistent with the findings of Zheng et al. [32]; however, the results of further interactive tests in this study suggest that this finding is not significant (*P*-interaction > 0.05). In addition to examining the correlation between pre-diabetes and TyG-BMI in people with a family history of diabetes, we also conducted an exploratory analysis in different age, sex, and BMI groups. Our evidence suggests that the TyG-BMI-related pre-diabetes risk was significantly higher in people younger than 50 years of age, women, and non-obese individuals. This finding is similar to the findings of subgroups of several recent TyG-BMI-related studies. In two recent TyG-BMI-related studies, some scholars noted that TyG-BMI was related to a significantly higher risk of NAFLD and diabetes in these populations [18, 19]. These findings suggest a special population dependence on the association of TyG-BMI with various metabolic diseases, among which more attention should be given to the evaluation of TyG-BMI in young and middle-aged people, women and non-obese people. Currently, the reasons for the specific population dependence of the association of TyG-BMI with multiple metabolic diseases are not clear, but the following points may require attention: (1) With the rapid development of society, urbanization and informatization, on-duty workers inevitably reduce their demand for physical activity, so they are more prone to metabolic problems [36]. (2) With the accelerated development of social informatization, young people have developed increasingly unhealthy living habits, which leads to a variety of metabolic problems prematurely [37, 38]. (3) Men and women have different body compositions; generally speaking, women have more fat mass, men have more lean mass [39], and BMI does not seem to reflect the obesity information of the body very well [40]. In addition, Asian people are also prone to secondary metabolic problems, even if they are non-obese [41].

Notably, the mean age of the subjects in this study was relatively young. Some previous studies have shown that abnormalities in lipoprotein cholesterol, apolipoprotein and lipoprotein (a) are closely related to premature cardiovascular disease [42–44], among which IR is the main pathological mechanism leading to susceptibility to premature cardiovascular disease [45]. TyG-BMI is a compound index including glucose and lipids and has excellent IR recognition ability. Combined with the findings of this study and the age characteristics of the subjects, we believe that TyG-BMI may also be used to predict the risk of premature cardiovascular disease.

### Study strengths and limitations

This study has several strengths worth mentioning: (1) The sample size of this study is more than 100,000 and involves many regions in China, and the conclusion of the study is relatively objective and applicable to the Chinese population. (2) The study was analysed based on two different diagnostic criteria and reached a consistent conclusion, which can be considered relatively reliable. (3) The discovery of special population dependence on the risk of TyG-BMI-related metabolic diseases provides a new idea for health surveillance in the general population.

Some limitations need to be emphasized in this study: (1) The diagnostic criteria for pre-diabetes in this study were based on IFG; however, in the Asian population, the use of isolated IFG for pre-diabetes appears to result in a lower prevalence [46]. Considering that there is no unified diagnostic standard for pre-diabetes at present, this study also demonstrated the relationship between pre-diabetes and TyG-BMI at a lower prevalence rate, further implying that the conclusions of this study are relatively stable. (2) Due to the retrospective design of this study, in addition to some inherent statistical limitations, the historical data that our researchers were able to extract were also relatively limited. For example, this study lacked general measurement information such as waist circumference, neck circumference and hip circumference as well as information about chronic diseases and drug use of the subjects. Therefore, we were unable to evaluate the predictive performance of these simple measurement parameters for pre-diabetes in this study, and we were also unable to perform some subgroup analyses of comorbidities and drug therapy. (3) The follow-up period of this study was relatively short, and further follow-up studies are needed to further explore the causal association between the two. (4) According to the results of previous studies, IR may be an important factor in the association of TyG-BMI with pre-diabetes [15]. However, relevant IR information was not measured in this study; thus, the mechanism of the association between pre-diabetes and TyG-BMI needs to be verified by further studies. (5) As the present study was conducted in the Chinese population, whether TyG-BMI can be used to assess the risk of pre-diabetes in other ethnic groups needs further study.

## Conclusion

Overall, TyG-BMI is independently associated with pre-diabetes, whether using the ADA or WHO diagnostic criteria. TyG-BMI may be an accessible and supplementary monitoring method in the risk stratification management of patients with pre-diabetes. In addition, there is a special population dependence between TyG-BMI and many metabolism-related diseases. Therefore, we suggest that TyG-BMI screening should be performed routinely in non-obese people, women and people under 50 years old.

## Supplementary Information

**Additional file 1.**

## Data Availability

The datasets that support the conclusions of this article can be found in the Dryad repository.

## Abbreviations

TyG-BMI: Triglyceride glucose-body mass index
ADA: American Diabetes Association
WHO: World Health Organization
IFG: Impaired fasting glucose
FPG: Fasting plasma glucose
IR: Insulin resistance
HEC: Hyperinsulinemic-euglycemic clamp
AUROC: Area under the receiver operating characteristic curve
HR: Hazard ratios
CI: Confidence intervals
KM: Kaplan-Meier
BMI: Body mass index
TC: Total cholesterol
TG: Triglyceride
LDL-C: Low density lipoprotein cholesterol
ALT: Alanine aminotransferase
AST: Aspartate aminotransferase
BUN: Blood urea nitrogen
Cr: Creatinine
HDL-C: High density lipoprotein cholesterol
TyG index: Triglyceride glucose index
MS: Metabolic syndrome
ROC: Receiver operating characteristic curve
NAFLD: Nonalcoholic fatty liver disease
