# Supplementary material for: Triglyceride glucose-body mass index in identifying high-risk groups of pre-diabetes
Source: Lipids Health Dis. 2021 Nov 14;20:161. doi: 10.1186/s12944-021-01594-7 (PMC8590771; doi:10.1186/s12944-021-01594-7)
Supplement: Supplementary file 1 — Additional file 1. [file 12944_2021_1594_MOESM1_ESM.docx]

Supplementary Table 1: Collinearity diagnostics steps.

|  | VIF | | | | |
| --- | --- | --- | --- | --- | --- |
|  | Step 1 | Step 2 | Step 3 | Step 4 | Step 5 |
| ADA |  |  |  |  |  |
| TyG-BMI | 446.5 | 138.4 | 2.5 | 2.5 | 2.2 |
| Age, years | 1.4 | 1.4 | 1.4 | 1.4 | 1.4 |
| Sex | 3.3 | 3.3 | 3.3 | 3.3 | 3.2 |
| Height, cm | 54 | 44.6 | 2.1 | 2.1 | 2.1 |
| Weight, kg | 171.8 | 141.2 | NA | NA | NA |
| BMI, kg/m^2^ | 314.7 | NA | NA | NA | NA |
| SBP, mmHg | 2.1 | 2.1 | 2.1 | 2.1 | 2.1 |
| DBP, mmHg | 2 | 2 | 2 | 2 | 2 |
| FPG, mmol/L | 1.2 | 1.2 | 1.2 | 1.2 | 1.1 |
| TyG index | 61.2 | 23.7 | 6.6 | 6.6 | NA |
| TC, mmol/L | 7.3 | 7.3 | 7.3 | NA | NA |
| TG, mmol/L | 5.7 | 5.2 | 5 | 4.7 | 1.7 |
| HDL-C, mmol/L | 1.6 | 1.6 | 1.6 | 1.2 | 1.2 |
| LDL-C, mmol/L | 6 | 6 | 6 | 1.2 | 1.1 |
| ALT, U/L | 3.3 | 3.3 | 3.3 | 3.3 | 3.3 |
| AST,U/L | 3 | 3 | 2.9 | 2.9 | 2.9 |
| BUN, mmol/L | 1.2 | 1.2 | 1.2 | 1.2 | 1.2 |
| Crr, umol/L | 2.1 | 2.1 | 2.1 | 2.1 | 2.1 |
| Family history of diabetes | 1 | 1 | 1 | 1 | 1 |
| Smoking status | 3.1 | 3.1 | 3.1 | 3.1 | 3.1 |
| Drinking status | 3.1 | 3.1 | 3.1 | 3.1 | 3.1 |
| WHO |  |  |  |  |  |
| TyG-BMI | 434.5 | 135.4 | 2.6 | 2.6 | 2.2 |
| Age, years | 1.4 | 1.4 | 1.4 | 1.4 | 1.4 |
| Sex | 3.2 | 3.2 | 3.2 | 3.2 | 3.2 |
| Height, cm | 52.5 | 43.1 | 2.1 | 2.1 | 2.1 |
| Weight, kg | 166.4 | 136 | NA | NA | NA |
| BMI, kg/m^2^ | 304.9 | NA | NA | NA | NA |
| SBP, mmHg | 2.1 | 2.1 | 2.1 | 2.1 | 2.1 |
| DBP, mmHg | 2 | 2 | 2 | 2 | 2 |
| FPG, mmol/L | 1.3 | 1.3 | 1.3 | 1.3 | 1.1 |
| TyG index | 60.7 | 23.8 | 6.8 | 6.8 | NA |
| TC, mmol/L | 7.1 | 7.1 | 7.1 | NA | NA |
| TG, mmol/L | 5.8 | 5.3 | 5.1 | 4.8 | 1.7 |
| HDL-C, mmol/L | 1.5 | 1.5 | 1.5 | 1.2 | 1.2 |
| LDL-C, mmol/L | 5.9 | 5.9 | 5.9 | 1.2 | 1.1 |
| ALT, U/L | 3.3 | 3.3 | 3.3 | 3.3 | 3.3 |
| AST, U/L | 3 | 3 | 2.9 | 2.9 | 2.9 |
| BUN, mmol/L | 1.2 | 1.2 | 1.2 | 1.2 | 1.2 |
| Cr, umol/L | 2.1 | 2.1 | 2.1 | 2.1 | 2.1 |
| Family history of diabetes | 1 | 1 | 1 | 1 | 1 |
| Smoking status | 3.1 | 3.1 | 3.1 | 3.1 | 3.1 |
| Drinking status | 3.1 | 3.1 | 3.1 | 3.1 | 3.1 |

Abbreviation: VIF: variance inflation factor; VIF = 1/(1-R^2^). Abbreviations as in Table ​1.

Note: The variables with VIF>5 will be regarded as collinear variables and cannot be included in the multiple regression model.
